# Colorectal cancer screening with fecal occult blood test: A 22-year cohort study

**DOI:** 10.3892/ol.2013.1402

**Published:** 2013-06-14

**Authors:** PENG JIN, ZI-TAO WU, SHI-RONG LI, SHU-JUN LI, JI-HENG WANG, ZHI-HONG WANG, JIAN-GUO LU, XIN-JUAN CUI, YING HAN, JIANYU RAO, JIAN-QIU SHENG

**Affiliations:** 1 Department of Gastroenterology, Beijing Military General Hospital, Beijing 100700, P.R. China; 2 Third Military Medical University, Chongqing 400038, P.R. China;; 3 Department of Pathology and Laboratory Medicine, David Geffen School of Medicine, P.R. China; 4 Department of Epidemiology, School of Public Health, University of California, Los Angeles, CA 90015, USA

**Keywords:** colorectal cancer, screening, fecal occult blood test

## Abstract

The aim of the present study was to investigate the efficacy of colorectal cancer (CRC) screening with a three-tier fecal occult blood test (FOBT) in the Chinese population. The study was performed between 1987 and 2008 at the Beijing Military General Hospital, in a cohort of army service males and females aged >50 years. Between 1987 and 2005, a three-tier screening program, comprising guaiac-based FOBTs (gFOBTs), followed by immunochemical FOBTs for positive guaiac test samples and then colonoscopy for positive immunochemical test subjects, was performed annually. The cohort was followed up until 2008. The cohort included 5,104 subjects, of which, 3,863 subjects participated in screening (screening group) and 1,241 did not (non-screening group). The two groups did not differ in age, gender or other major risk factors for colon cancer. Overall, 36 CRCs occurred in the screening group and 21 in the non-screening group. Compared with the non-screening group, the relative risk for the incidence and mortality of CRC was 0.51 [95% confidence interval (CI), 0.30–0.87] and 0.36 (95% CI, 0.18–0.71), respectively, in the screening group. The general sensitivity of this three-tier FOBT was 80.6% (95% CI, 65.3–91.1). Thus, annual screening using the three-tier FOBT program may reduce the CRC incidence and mortality rate.

## Introduction

Colorectal cancer (CRC) is the second most common type of cancer and the second leading cause of cancer-related mortality in developed countries (1). In recent years, the incidence of CRC has markedly increased in China, particularly in urban populations. This is likely to be due to the rapid changes in social economic factors, such as lifestyle, diet and environment. As an example, in Beijing, the annual incidence of CRC has increased from 16 per 100,000 to 24 per 100,000 in the past decade (2).

Screening provides the ability to detect precancerous lesions and early-stage CRC, thus saving lives, and there are numerous different screening options presently available (3). Among these, the fecal occult blood test (FOBT) is the most common test in use for CRC screening. Several large cohort studies have demonstrated the effectiveness of FOBT-based screening in Western countries (4–6); however, no such studies have been performed in developing countries thus far. Furthermore, the majority of the large-scale cohort studies have utilized the conventional chemical guaiac-based FOBT (gFOBT) method. The gFOBT has a high false-positive rate (range, 2–13%) (7,8) and other limitations, such as the requirement of diet restriction. The latter is particularly troublesome for people in Asian countries. The antibody-based immunochemical method, the fecal immunochemical test (FIT), has been demonstrated to have test performance characteristics with improved sensitivity and specificity compared with those of gFOBT, and it does not require any restrictions on diet (7). However, the higher costs associated with FIT compared with gFOBT limit its use for screening in countries such as China, where healthcare resources are limited. The present study evaluates a three-tier FOBT-based program, and presents the results of a prospective longitudinal trial for CRC screening with this program.

## Materials and methods

### Subjects

The eligible subjects in the present study were army officers (retired or non-retired) aged >50 years, who were receiving healthcare from the Beijing Military General Hospital (Beijing, China) and lived in the Beijing area. Subjects with known CRC, colorectal adenomas, inflammatory bowel disease or various types of malignant tumors were excluded from the cohort. The study was approved by the Beijing Military General Hospital Ethics Committee, and informed consent was obtained from each subject.

The present study was a dynamic cohort study, where subjects who met the eligibility criteria were recruited on an annual basis. Upon entry into the cohort study, each subject received a complete health status check-up, which included a physical examination, chest X-ray, ECG, abdominal ultrasound, mammogram (for females) and serological examination, including an analysis of glucose and lipid levels. The health check-up data, along with the data from a baseline questionnaire (for date of birth, gender, education, family history of malignant tumors, lifestyle, medication use, body height/weight and previous general health status), were entered into a database. The database was updated yearly, based on the findings from the annual follow-up examinations.

At the end of the initial check-up, each eligible subject was asked by physicians for their consent to undergo CRC screening. Those who agreed to be screened were included in the screening group, and those who refused to be screened were included in a non-screening group. Other than the FOBT that was performed in the screening group on an annual basis, the subjects in the screening and non-screening groups were investigated in the same manner, with an annual examination for other chronic conditions, as detailed above.

**Figure 1.**
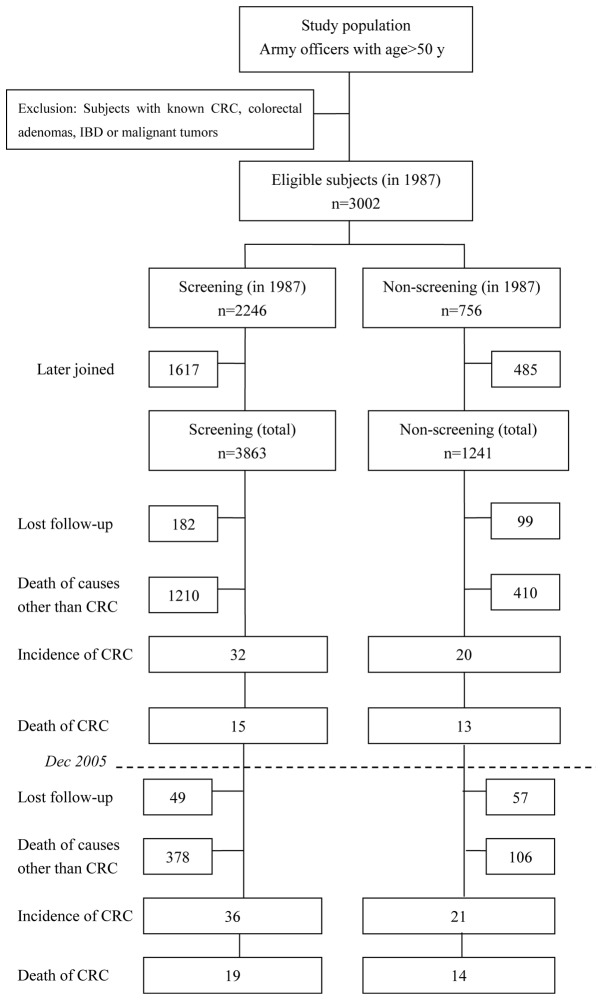
Diagram depicting the screened versus non-screened population and their outcomes.

The annual FOBT-based CRC screening began in May 1987 and ended in December 2005. Following this, the two groups were followed up until December 2008. In 1987, there were 3,002 eligible subjects, including 2,260 males and 742 females, with a mean age of 61.8 years (2,809 subjects were aged ≤74 years). The number of subjects in the screening group was 2,246, and the number of subjects in the non-screening group was 756. For the subsequent years (1988–2005), an additional 2,102 eligible subjects were entered into the cohort. In total, there were 3,863 subjects in the screening group and 1,241 in the non-screening group (Fig. 1).

### Screening methods

For the screening, each subject provided one fecal sample every year for the FOBT, which was performed in the Clinical Laboratory of Beijing Military General Hospital. No restrictions, with regard to diet, drugs or other food items, were imposed on the subjects prior to stool collection. Between 1987 and 2005, a three-tier screening program was implemented: gFOBT (rehydrated; produced by Baso Diagnostics Inc., Zhuhai, China) was performed, followed by FIT (gold gel stripe; provided by WanhuaPuman Biological Engineering Co., Ltd., Tech Lt. Comp., Beijing, China) for gFOBT-positive fecal samples, and then colonoscopy for those who had positive FIT results. According to the manufacturer, the positive threshold of FIT was 0.2 *μ*g/ml. For patients who refused colonoscopy, a double-contrast barium enema (DCBE) was performed. For patients with negative (no cancer detected) colonoscopy or DCBE results, the aforementioned screening steps were repeated in the following year.

On identification of a lesion (polyp, tumor or otherwise) during colonoscopy or DCBE, a biopsy (or resection for a polyp or tumor) was performed. If a malignant tumor was identified, appropriate management was provided according to the final pathology, and the subject was regarded as an incidence case (the secondary endpoint) and continued to be followed up until they succumbed to the disease (the primary endpoint). Patients with adenoma were to be followed annually, as were the other non-cancer subjects, following complete resection.

### Data and statistical analyses

The primary endpoint of the follow up was CRC-related mortality, and the secondary endpoint was the incidence of CRC. Overall, 82% of the mortalities occurred in the Beijing Military General Hospital, and the cause was determined based on a review of the medical records (International Classification of Diseases, ICD-9 or -10). For the subjects who succumbed elsewhere, a telephone interview with the next of kin was performed and the cause of death was determined from the death certificate.

A positive predictive value (PPV) was estimated for each screening round, while only positive tests followed by colonoscopy or DCBE were used in the computation. The general sensitivity was evaluated by the number of true positives relative to the number of individuals with carcinomas, while a positive test result was considered to be a true positive if a carcinoma was detected during the entire follow-up period.

To evaluate potential selection bias, the χ^2^ test or the independent samples t-test for the baseline characteristics [age at baseline, gender, education, family history of malignant tumors, body mass index (BMI), smoking status (never, former or current), alcohol intake (none, occasional or regular), physical exercise (at least once per week or less), meat intake (at least three times per week or less) and aspirin use (regular or never)] and status at time of mortality from all of these causes were utilized. A χ^2^ test was used to examine the CRC incidence and mortality in the screening and non-screening groups, and the relative risk (RR) and 95% confidence intervals (CIs) were calculated. The Kaplan-Meier survival analysis was used to evaluate the effect of screening on CRC incidence and mortality, and the cumulative incidence and cumulative mortality curves were determined. The Cox proportional hazards regression model was used to control potential confounding factors (age at baseline, gender, education, family history of malignant tumors, BMI, smoking status, alcohol intake, physical exercise, meat intake and aspirin use) for CRC incidence and mortality. SPSS 13.0 software (SPSS, Inc., Chicago, IL, USA) was used for the statistical analyses. All statistical tests that were performed were two-sided, and P<0.05 was considered to indicate a statistically significant difference.

## Results

### Subjects and screening results

Fig. 1 shows a diagram depicting the screened vs. non-screened population and the outcome of the study. For the entire 22-year study period, there were a total of 5,104 eligible subjects. Of these, 3,863 participated in the screening for CRC (screening group) and 1,241 did not (non-screening group). Over the course of the study, 231 subjects in the screening group and 156 in the non-screening group were lost to follow-up due to a change in residence (6.0 and 12.6%, respectively; P=0.000). In addition, 1,588 subjects in the screening group and 516 in the non-screening group had succumbed due to causes other than CRC (41.1 and 41.6%, respectively; P=0.769). Up to December, 2008, the person-years of observation in the screening and non-screening groups were 49,566 and 15,826, respectively. The baseline characteristics of the subjects were not significantly different in the screening group compared with those in the non-screening group (Table I).

Table II shows the results of each screening round. The positive result rates of gFOBT ranged from 4.6 to 16.2%, while FIT ranged from 1.1 to 2.6%. Between 1987 and 2005, 778 colonoscopies and 33 DCBEs were performed in the screening group, with a total follow-up rate of 87.0% (811/932) in patients with positive FIT results. A total of 36 cases of CRC occurred in the screening group. Among the 36 CRC cases, 25 were detected by the screening program, while four cases with positive FIT results refused to undergo follow-up colonoscopy or DCBE, and were later diagnosed by clinical means. Seven cases were missed due to a false-negative FOBT result. The general sensitivity of this three-tier FOBT was 80.6% (29/36; 95% CI, 65.3–91.1). There were 153 (4.0%) subjects who were identified to have adenomas of ≥1 cm in size in the screening group.

### Incidence and mortality rates of CRC in screening and non-screening groups

During the 19-year screening period (1987–2005), 32 CRC cases occurred in the screening group and 20 occurred in the non-screening group. In the last 3 years of follow-up (2006–2008), an additional four cases occurred in the screening group and one occurred in the non-screening group (Fig. 1 and Table III). Until December 2005, the CRC incidence in the screening group was 0.75/1,000 person-years, which was significantly lower than that of the non-screening group (1.43/1,000 person-years; P=0.027). The mortality was 0.35/1,000 person-years in the screening group, which was also significantly lower than that of the non-screening group (0.93/1,000, P= 0.013). The relative risks of incidence and mortality adjusted for the baseline characteristics of the subjects were 0.49 (95% CI, 0.28–0.85) and 0.31 (95% CI, 0.14–0.65), respectively (Both P<0.05; Table III). Three years subsequent to the termination of the screening program, the incidence and mortality in the screening group was significantly lower than that of the non-screening group (P=0.034 and P=0.022, respectively), with adjusted relative risks of 0.51 (95% CI, 0.30–0.87) and 0.36 (95% CI, 0.18–0.71), respectively (both P<0.05; Table III). Therefore, there was a 49% decrease in incidence and a 64% decrease in mortality in the screening group compared with the non-screening group during the entire 22-year study period. Mortality rates from all causes did not differ between the two groups (P=0.516 for between 1987 and 2008).

In total, the study was comprised of 4,777 subjects aged between 50 and 74 years, and 327 subjects aged >75 years on recruitment. A stratified analysis revealed that a significant decrease in the incidence and mortality of CRC was only evident in the subgroup who were aged between 50 and 74 years (P=0.028 and P=0.024, respectively; Table IV).

**Figure 2.**
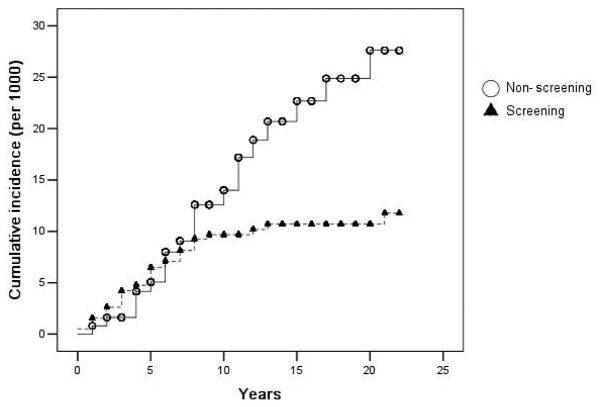
Cumulative incidence of colorectal cancer (CRC) in the screening and non-screening group by Kaplan-Meier analysis.

**Figure 3.**
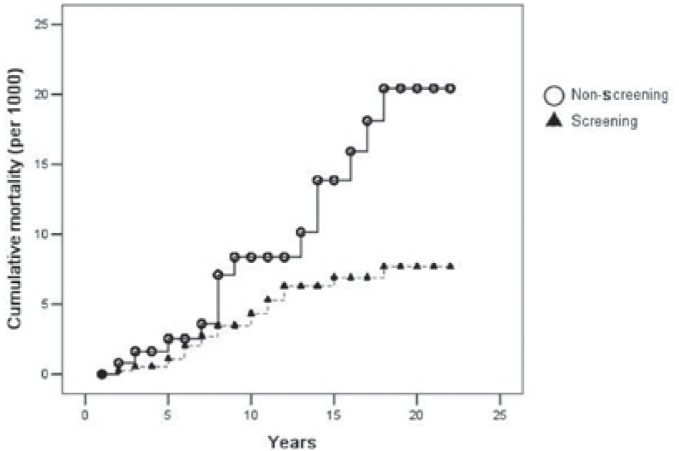
Cumulative mortality of colorectal cancer (CRC) in the screening and non-screening groups, by Kaplan-Meier analysis.

Figs. 2 and 3 show the cumulative incidence and mortality of CRC, as analyzed by the Kaplan-Meier method. The differences in the cumulative incidence and mortality between the two groups was evident after the seventh and eighth years of screening, and more so thereafter.

## Discussion

The current study presented a three-tier FOBT-based screening program, which has been described previously (9,10). Certain cross-sectional studies for this type of combined gFOBT with FIT have demonstrated compatible sensitivity to gFOBT, but notable increased specificity (7,10). The difficulty of dietary restriction led to a poor specificity of guaiac-based tests in the Chinese population (11). Moreover, the advantage of using a combined test is that it reduces the cost of the FIT assay, as the FIT is only developed when the gFOBT result is positive. The observed sensitivity for CRC detection in the present study was substantially greater than that identified by Allison *et al* (8) (65% for CRC) in a cross-sectional study with a similar three-tier method. This is likely to be due to the longitudinal nature of the present study, which meant that the cases missed in one screening round were identified in subsequent repeat tests.

In this 22-year longitudinal, controlled trial involving a dynamic cohort, it was demonstrated that annual FOBT-based screening resulted in a 49% decrease in colon cancer incidence and a 64% decrease in CRC-related mortality, even 3 years after the termination of the screening program. The differences in cumulative incidence and mortality were evident between the two groups after only 7 to 8 years of the study, which may indicate that the lead time of screening is ∼7–8 years. To the best of our knowledge, this study is the longest follow-up of a colon cancer screening trial in a developing country. The study included a highly stable cohort with relatively few subjects lost to follow-up (<10% in the 22-year follow-up), but a high censoring rate due to mortality from other causes. The compliance rate was also high (almost 100% for the first level test and 83% for colonoscopy in patients with a positive FIT result). This may be ascribed partly to bringing the screening program to a military healthcare system.

However, the study also has several limitations. The population of the study consisted only of military officials and may not consequently be representative of the general Chinese population. Additionally, the study was not randomized; the screening group consisted of subjects who chose to be screened. This may have produced a selection bias, although the screened and non-screened populations shared similar demographic features. Furthermore, there is no definite final age for the screening. Routine screening for CRC is recommended against in adults >75 years (12). The present data also revealed that the effect of screening on the incidence and mortality of CRC was less significant among subjects aged >75 years. Moreover, the sample size was relatively small compared with previous trials involving gFOBT.

Taking into account the aforementioned limitations, the present study demonstrated that a three-tier FOBT-based annual screening, even with one fecal sample, resulted in the detection of >80% of CRCs. The study also revealed a marked decrease in CRC-related mortality (64% after controlling potential confounding factors) compared with that which has been identified previously with gFOBT, which was in the range of 15 to 30% (4–6). The present findings were similar to the longitudinal non-controlled observational study in the Japanese population by Lee *et al* (13), which utilized FIT for screening and demonstrated a 70% decrease in mortality over a 13-year study period. Moreover, there are a limited number of studies demonstrating an effect of FOBT on incidence (14), while the present data revealed a 49% decrease in CRC incidence in the screening group compared with that in the non-screening group. This dramatic decrease in the incidence and mortality of CRC may be ascribed partly to the higher compliance and adenoma detection rates with subsequent resection in the present study. In the study 4% of the screening subjects were identified to have adenomas of ≥1 cm in size, while 0.8–1.7% were identified in a previous study (15). However, since the present study was not randomized, the effect of selection bias on the differences in incidence and mortality observed may not be excluded, although the two groups (screening and non-screening) exhibited similar baseline demographic characteristics. Furthermore, the grouping in the present study was according to the preference of the subjects, i.e. whether to undergo CRC screening or not. It is supposed that individuals who refuse screening have higher CRC incidence and mortality rates than those who accept testing (16). The rates of loss to follow-up were significantly higher in the non-screening group, which may indicate less benefit from the military healthcare system in the non-screening group compared with that in the screening group. All of the aforementioned factors may have contributed to the decreases in the incidence and mortality of CRC, along with the efficacy of the screening process.

In conclusion, when accepted, annual FOBT-based screening using a three-tier system in an average-risk Chinese population aged >50 years demonstrated >80% sensitivity in detecting CRC. The screening significantly reduced the incidence and mortality rates of CRC. These findings suggested that annual FOBT examination coupled with a complete colonoscopy follow-up for cases with positive FOBT results is an effective approach for CRC control in China.

## Figures and Tables

**Table I.** Baseline characteristics of subjects in the screening and non-screening groups.

Characteristics	Screening (n=3863)	Non-screening (n=1241)	P-value
Gender, n (%)			
Male	2913 (75.4)	934 (75.3)	0.917
Female	950 (24.6)	307 (24.7)	
Age (years)			
Mean age ± SD	62.1±6.9	62.0±7.0	0.613
Education, n (%)			
Up to high school	3549 (91.9)	1133 (91.3)	0.523
College or higher	314 (8.1)	108 (8.7)	
Family history of malignant tumors, n (%)			
With	118 (3.1)	31 (2.5)	0.311
Without	3745 (96.9)	1210 (97.5)	
Body mass index			
Mean BMI ± SD	23.1±1.6	23.2±1.6	0.736
Smoking status, n (%)			
Never	1339 (34.7)	406 (32.7)	0.082
Former	1120 (29.0)	401 (32.3)	
Current	1404 (36.3)	434 (35.0)	
Alcohol intake, n (%)			
None	747 (19.3)	244 (19.7)	0.630
Occasional	2810 (72.7)	909 (73.2)	
Regular	306 (7.9)	88 (7.1)	
Physical exercise, n (%)			
<Once per week	1853 (48.0)	593 (47.8)	0.910
≥Once per week	2010 (52.0)	648 (52.2)	
Meat intake, n (%)			
<3 times per week	1038 (26.9)	331 (26.7)	0.891
≥3 times per week	2825 (73.1)	910 (73.3)	
Aspirin, n (%)			
No use	2957 (76.5)	958 (77.2)	0.638
Regular use	906 (23.5)	283 (22.8)	

**Table II.** Results of each annual screening round and the test performance of FOBT.

Year	No. of subjects	Screened subjects		gFOBT^+^		FIT^+^		Colonoscopy or DCBE		Cancer		Cancer and adenomas ≥1 cm	
		n	%	n	%	n	%	n	%^a^	n	PPV^b^	n	PPV^b^
1987	2246	2246	100.0	202	9.0	51	2.3	45	88.2	1	2.2	10	22.2
1988	2239	2233	99.7	175	7.8	48	2.1	39	81.3	1	2.6	8	20.5
1989	2240	2228	99.5	237	10.6	52	2.3	47	90.4	2	4.3	8	17.0
1990	2211	2205	99.7	156	7.1	47	2.1	35	74.5	0	0.0	7	20.0
1991	2184	2164	99.1	233	10.8	52	2.4	45	86.5	1	2.2	12	26.7
1992	2286	2253	98.6	254	11.3	55	2.4	49	89.1	2	4.1	8	16.3
1993	2293	2285	99.7	216	9.5	54	2.4	46	85.2	1	2.2	11	23.9
1994	2264	2251	99.4	365	16.2	58	2.6	53	91.4	2	3.8	10	18.9
1995	2308	2302	99.7	269	11.7	61	2.6	57	93.4	3	5.3	15	26.3
1996	2280	2247	98.6	271	12.1	54	2.4	51	94.4	2	3.9	11	21.6
1997	2220	2214	99.7	204	9.2	52	2.3	46	88.5	1	2.2	7	15.2
1998	2217	2201	99.3	316	14.4	50	2.3	44	88.0	1	2.3	12	27.3
1999	2255	2249	99.7	198	8.8	53	2.4	39	73.6	1	2.6	9	23.1
2000	2253	2236	99.2	215	9.6	52	2.3	50	96.2	1	2.0	14	28.0
2001	2232	2218	99.4	192	8.7	48	2.2	43	89.6	1	2.3	5	11.6
2002	2289	2273	99.3	183	8.1	45	2.0	41	91.1	1	2.4	11	26.8
2003	2457	2415	98.3	112	4.6	26	1.1	21	80.8	1	4.8	6	28.6
2004	2457	2445	99.5	227	9.3	48	2.0	36	75.0	2	5.6	6	16.7
2005	2456	2431	99.0	145	6.0	26	1.1	24	92.3	1	4.2	8	33.3

a Rates were the number of colonoscopy or double-contrast barium enema (DCBE) patients relative to the number of patients with positive fecal immunochemical test (FIT) results.

b Only positive tests followed by colonoscopy or DCBE were used in the computation for positive predictive value (PPV). gFOBT, guaiac-based fecal occult blood test.

**Table III.** Incidence and mortality rates of CRC between 1987 and 2008 (per 1000 person-years).

Characteristic	May 1987-December 2005		May 1987-December 2008	
	Screening	Non-screening	Screening	Non-screening
Person-years of observation	42881	13974	49566	15826
Colorectal cancer				
No. of patients	32	20	36	21
Incidence rate	0.75	1.43	0.73	1.33
RR (95% CI)	0.52 (0.30–0.93)		0.55 (0.32–0.95)	
Adjusted RR (95% CI)^a^	0.50 (0.29–0.88)		0.52 (0.30–0.89)	
Adjusted RR (95% CI)^b^	0.49 (0.28–0.85)		0.51 (0.30–0.87)	
Mortality from CRC				
No. of deaths	15	13	19	14
Mortality rate	0.35	0.93	0.38	0.88
RR (95% CI)	0.38 (0.18–0.81)		0.43 (0.22–0.88)	
Adjusted RR (95% CI)^a^	0.31 (0.15–0.67)		0.36 (0.18–0.72)	
Adjusted RR (95% CI)^b^	0.31 (0.14–0.65)		0.36 (0.18–0.71)	
Mortality from all causes				
No. of deaths	1225	423	1607	530
Mortality rate	28.57	30.27	32.42	33.49
RR (95% CI)	0.94 (0.85–1.05)		0.97 (0.88–1.07)	

a Adjusted for age and gender.

b Adjusted for age, gender, education, family history of malignant tumors, BMI, smoking status, alcohol intake, physical exercise, meat intake and aspirin use. CRC, colorectal cancer; RR, relative risk; CI, confidence interval.

**Table IV.** Incidence and mortality of CRC stratified by age (between May, 1987 and December, 2008).

	50–74 years		≥75 years	
	Screening	Non-screening	Screening	Non-screening
No. of subjects	3614	1163	249	78
No. of CRC cases	29 (0.8%)	18 (1.5%)	7 (2.8%)	3 (3.8%)
RR (95% CI)	0.52 (0.29–0.93)		0.71 (0.18–2.74)	
Adjusted RR (95% CI)^a^	0.50 (0.28–0.90)		0.69 (0.18–2.67)	
Mortality from CRC	14 (0.4%)	11 (0.9%)	5 (2.0%)	3 (3.8%)
RR (95% CI)	0.40 (0.18–0.89)		0.43 (0.10–1.83)	
Adjusted RR (95% CI)^a^	0.36 (0.16–0.80)		0.41 (0.10–1.74)	

a Adjusted for age and gender. CRC, colorectal cancer; RR, relative risk; CI, confidence interval.
